# Effects of Seclusion and Restraint in Adult Psychiatry: A Systematic Review

**DOI:** 10.3389/fpsyt.2019.00491

**Published:** 2019-07-16

**Authors:** Marie Chieze, Samia Hurst, Stefan Kaiser, Othman Sentissi

**Affiliations:** ^1^Adult Psychiatry Division, Department of Psychiatry, University Hospital of Geneva, Geneva, Switzerland; ^2^Institute for Ethics, History and the Humanities, Faculty of Medicine, University of Geneva, Geneva, Switzerland

**Keywords:** coercion, restraint, seclusion, psychiatry, inpatient, effect, safety, effectiveness

## Abstract

**Background:** Determining the clinical effects of coercion is a difficult challenge, raising ethical, legal, and methodological questions. Despite limited scientific evidence on effectiveness, coercive measures are frequently used, especially in psychiatry. This systematic review aims to search for effects of seclusion and restraint on psychiatric inpatients with wider inclusion of outcomes and study designs than former reviews.

**Methods:** A systematic search was conducted following PRISMA guidelines, primarily through Pubmed, Embase, and CENTRAL. Interventional and prospective observational studies on effects of seclusion and restraint on psychiatric inpatients were included. Main search keywords were *restraint*, *seclusion*, *psychiatry*, *effect*, *harm*, *efficiency*, *efficacy*, *effectiveness*, and *quality of life*.

**Results:** Thirty-five articles were included, out of 6,854 records. Studies on the effects of seclusion and restraint in adult psychiatry comprise a wide range of outcomes and designs. The identified literature provides some evidence that seclusion and restraint have deleterious physical or psychological consequences. Estimation of post-traumatic stress disorder incidence after intervention varies from 25% to 47% and, thus, is not negligible, especially for patients with past traumatic experiences. Subjective perception has high interindividual variability, mostly associated with negative emotions. Effectiveness and adverse effects of seclusion and restraint seem to be similar. Compared to other coercive measures (notably forced medication), seclusion seems to be better accepted, while restraint seems to be less tolerated, possibly because of the perception of seclusion as “non-invasive.” Therapeutic interaction appears to have a positive influence on coercion perception.

**Conclusion:** Heterogeneity of the included studies limited drawing clear conclusions, but the main results identified show negative effects of seclusion and restraint. These interventions should be used with caution and as a last resort. Patients’ preferences should be taken into account when deciding to apply these measures. The therapeutic relationship could be a focus for improvement of effects and subjective perception of coercion. In terms of methodology, studying coercive measures remains difficult but, in the context of current research on coercion reduction, is needed to provide workable baseline data and potential targets for interventions. Well-conducted prospective cohort studies could be more feasible than randomized controlled trials for interventional studies.

## Introduction

### Rationale

Coercion is a theme of worldwide importance in psychiatry and is defined as the use of an intervention against a person’s will (1). Coercive measures can also have other dimensions, in particular, limitations of freedom of movement (1) that are frequently used in psychiatry, usually for containment of aggressive behaviors, but also in other circumstances and settings, including every medical specialty (2, 3). In the context of overriding a person’s will, coercion raises ethics and legal questions. These measures limit several fundamental human rights, such as liberty of choice or movement, autonomy, and physical integrity (4), and are therefore subjected to international, European, state, and local laws and regulations (5, 6). Discrepancies regarding the use of coercive measures between countries and even regions inside the same country are important (7–9). They concern clinical practices as well as juridical and ethical application of laws or recommendations. Efforts are made for an international harmonization of guidelines and practices, for example, through the “European Evaluation of Coercion in Psychiatry and Harmonization of Best Clinical Practice” (EUNOMIA) project (10, 11).

Various forms of coercion exist that can be differentiated into formal, informal, and subjective coercion, but their definitions and interpretations vary between countries (12). Formal coercion usually includes involuntary admission, involuntary treatment, seclusion, and restraint. The two latter categories refer to methods limiting freedom of physical movement. Several kinds of physical restraint exist either mechanical when devices are used for immobilization or manual when staff holds the patient. Seclusion is the confinement of the patient in a locked room from which he cannot exit on his own (1). Involuntary admission corresponds to the hospitalization of the patient against his will. Involuntary treatment refers to the administration of a medication against the will of the patient (1). The concept of this coercive measure is however very heterogeneous and can take several forms and definitions depending on the local or state legislations (5). Informal coercion regroups persuasion, manipulation, or other types of control or influence (12, 13). Subjective coercion characterizes patients’, caregivers’, or stakeholders’ points of view or feelings in situations of coercion. Subjective perception can differ from objective events (14).

The topic of coercion is particularly relevant in psychiatry as patients suffering from psychiatric disorders can lack decision-making capacity. The latter are thus susceptible to the other’s influence or power abuse (12). This susceptibility can lead to disrespect of human rights (15). In this context, use of coercive measures in psychiatry is controversial and needs to be practiced with great care (16).

Importantly, clinical practice should follow the principles of evidence-based medicine. By definition, an intervention is legitimate only if a direct benefit for the patient is scientifically proven (17). However, few data exist on the real benefit of coercive measures, regarding efficiency, efficacy, or effectiveness (18, 19). Several problems inherent to the topic limit application of rigorous scientific methodology. These problems include the heterogeneity of definitions (four formal types of coercion, informal and subjective types) and clinical practices (variation between countries and between hospital of the same country) (4) as well as difficulties in collecting valid and reliable data, with patients not always capable of consenting to research and randomization difficult to implement (20). Despite these scientific limitations in the evidence base, coercive measures are commonly used in adult psychiatric clinical practice. Some multicenter studies reported epidemiological data on the difference of use of coercion between countries or hospitals of the same country. Globally the rate of use of coercive measures in the literature varies from 0.4% to 66% (21). In a multicenter study in 10 German psychiatric hospitals, Steinert et al. reported an exposition to coercion in 9.5% of admissions (22). In Martin et al., 6.6% of admissions in seven Swiss psychiatric hospitals were affected by mechanical restraint compared to 10.4% of admissions in seven German hospitals (23). In the same study, 17.8% and 7.8% of admissions were respectively affected by seclusion. The EUNOMIA multicenter project conducted at 13 centers in 12 European countries studied the characteristics of the use of coercion and of the patient population submitted to coercive measures (seclusion, restraint, and/or forced treatment) and searched for differences between countries (11). The results showed significant variations of frequency of use between countries, from 21% to 59% of involuntary admissions, with higher rates in Poland, Italy, and Greece (8, 24). These discrepancies between lack of evidence for efficiency and frequency of use highlight the need for further study of the effects of coercion in adult psychiatry.

In addition, recent research has addressed coercion reduction, mainly through development of programs aiming to reduce coercive measures (3, 25). However, in order to evaluate the effectiveness of coercion reduction, objective data on baseline measures are needed without implementation of specific interventions to reduce coercion. For these reasons, studies on the consequences of coercive measures are of great scientific and clinical importance. Gutheil stated in 1978 that seclusion could in theory permit containment, reassurance, and diminution of sensible input (26). Several studies and reviews have since studied risk factors and effects of seclusion and restraint, but the results for effectiveness have been extremely limited (18, 19). Predictors of the use of coercive measures have been studied more extensively (27, 28) but mainly through retrospective databases and analyses (16). Sailas and Fenton (19) and Nelstrop et al. (29), two systematic reviews, and Luciano et al., a critical review (28) found two randomized controlled trials (RCT), but no other studies reporting results on effects or safety of seclusion or restraint with equivalent levels of evidence. Furthermore, the two systematic reviews have not been updated since 2012. The subject is of high importance due to the substantial consequences for patients, especially in case of severe mental disorders. In our view, one condition for legitimacy of coercive measures, not only juridical but also ethical and clinical, should be a beneficial effect for the patient. This could be a protective effect, but we were also concerned about the belief that coercive measures can have therapeutic effects. We have observed this belief in our experience, and it has also been described in the literature (30). We wanted to investigate whether or not this belief has scientific bases. An update of the recent literature on the evidence of efficiency (including efficacy, effectiveness and therapeutic benefit) of coercive measures is thus needed in order to evaluate the legitimacy of their use in clinical practice.

Due to the complexity of the subject, the systematic review needs to be limited to specific, well-defined questions. Efficacy of coercive measures is a fundamental question, as their use implies important clinical, ethical, and legal consequences. Concerning involuntary treatment, a direct beneficial and therapeutic effect seems more intuitive than for seclusion or restraint. Involuntary hospitalization is another way of coercion, for which the initial decision is mainly made outside of the hospital context. For these reasons, we chose to limit the present review to the study of seclusion and restraint that represent coercive measures limiting freedom of movement in order to investigate harmful or beneficial effects of these measures. Including involuntary treatment or hospitalization seemed to us to be another research question and would widen the scope of research questions too much for them to be answered in a single review. In addition, these methods directly concern the institutional practices in most countries and searching for their effectiveness and efficacy could provide important information for interventional studies aimed at seclusion and restraint reduction in clinical practice.

### Objectives and Research Question

The aim of this study is to conduct a systematic literature review on the negative and potentially beneficial effects of seclusion and restraint on adult psychiatric inpatients, compared to non-exposure or to exposure to other coercive measures. This review should permit establishing the potential harms and benefits of these measures and, therefore, provide an improved evidence base for making decisions in acute psychiatric care. In addition, through systematic synthetization of available baseline data, this review should provide arguments for later implementation of coercion reduction programs. Finally, we aim to synthesize the methods used to study the topic in order to propose a systematic approach for structuring research and improvement of the evidence basis.

As Sailas and Fenton found already in 2012, there have been only two randomized controlled trials on the effectiveness of seclusion and restraint (19). We chose to widen the search to prospective observational studies with various outcomes measuring benefits and harms of seclusion and restraint. Even though this approach limits the evidence level, it will allow for a broader appreciation of the consequences of interventions limiting liberty of movement.

## Material and Methods

### Study Design

This systematic review of the literature follows Preferred Reporting Items for Systematic Reviews and Meta-Analyses (PRISMA) guidelines, with a search question defined with the participants, interventions, comparators, outcomes, study design (PICOS) method as described in the Cochrane Collaboration Handbook (31). The studied population includes psychiatric inpatients hospitalized in adult psychiatric inpatient units. Interventions are seclusion and/or physical restraint (mechanical or manual). The comparator is either non-exposure to seclusion and/or restraint or exposure to other coercive measures [involuntary admission or treatment, or seclusion and/or restraint (the one that is not the main intervention)]. We considered a broad range of potential beneficial and negative effects of seclusion and restraint, including objective effectiveness (symptom intensity, level of needed medication, and length of stay), safety, adverse effects, quality of life, incidence of post-traumatic stress disorder (PTSD), and patients’ subjective perception of coercion.

### Eligibility Criteria

#### Inclusion Criteria

Articles studying adult psychiatric inpatients and physically limiting coercive measures (seclusion or restraint) were selected. We included interventional studies (including randomized controlled trials) and prospective observational studies including case-control studies. Articles published in English, French, or German were included. Articles investigating effects of seclusion and restraint on adult psychiatric inpatients were included. After full-text assessment, we synthesized the various studied outcomes and summarized them in different subgroups, which are detailed in **Table 1**: objective effectiveness (symptom intensity, level of needed medication, and length of stay), safety, adverse effects, quality of life, incidence of PTSD, and patients’ subjective perception of coercion.

**Table 1:** Explored outcomes.

Outcomes	Subgroups
Objective effectiveness	Symptoms intensity (including aggressiveness) (evaluated with PANSS (32, 33), BPRS (34–37) and BDI-II (36)) Need to change intervention Levels of needed medication Readmission rate Time to emergency resolution Length of stay Safety Quality of life after intervention Global functioning during and after intervention Ward environment
Adverse effects	Incidence of deep vein thrombosis during restraint Incidence of PTSD after intervention Influence of history of life-threatening events on traumatic effects of intervention Reported hallucinations during seclusion Occurrence of adverse events: agitation, suicide attempt or self-harm, revival of previous traumatism, death, hypertension, physical pain or fracture
Patients’ subjective perception	Positive and negative reported feelings during and after intervention Acceptance and comprehension of intervention (helpful, necessary, or disapproved) Level of perceived coercion Discrepancy between objective and reported coercion Evaluation of interaction and dialogue with staff Influence of ward environment on perceived coercion Feeling of improvement, safety, or security during and after intervention Preferences between different coercive measures

PANSS, Positive and Negative Syndrome Scale; BPRS, Brief Psychiatric Rating Scale; BDI-II, Beck Depression Inventory II.

#### Exclusion Criteria

Studies involving specific populations were excluded: non-psychiatric, geriatric, pediatric, outpatient, or forensic populations; somatic, addictive, or eating disorders; and intellectual disabilities. Studies on other coercive measures (involuntary admissions, forced medication, or informal coercion) were excluded.

We excluded retrospective studies (including extraction from databases), case series, and expert opinions. Qualitative studies restricted to thematic analyses were not included due to lack of objective data. Articles in other than the above-mentioned languages were not included. Studies on the staff’s attitude to seclusion or restraint were not included, as in most studies these are considered predictive factors, rather than effects, of coercive measures. Articles focused on risk factors or coercion reduction programs were excluded because they did not meet the search question criteria.

### Search Strategies

We searched the following databases: MEDLINE *via* Pubmed, Embase, Web of Science, PsycINFO, Google Scholar, Cochrane Central Register of Controlled Trials (CENTRAL), Cumulative Index to Nursing and Allied Health Literature (CINAHL) *via* EBSCO, Cairninfo, PROSPERO, and Clinicaltrials.gov.

**Figure 1:**
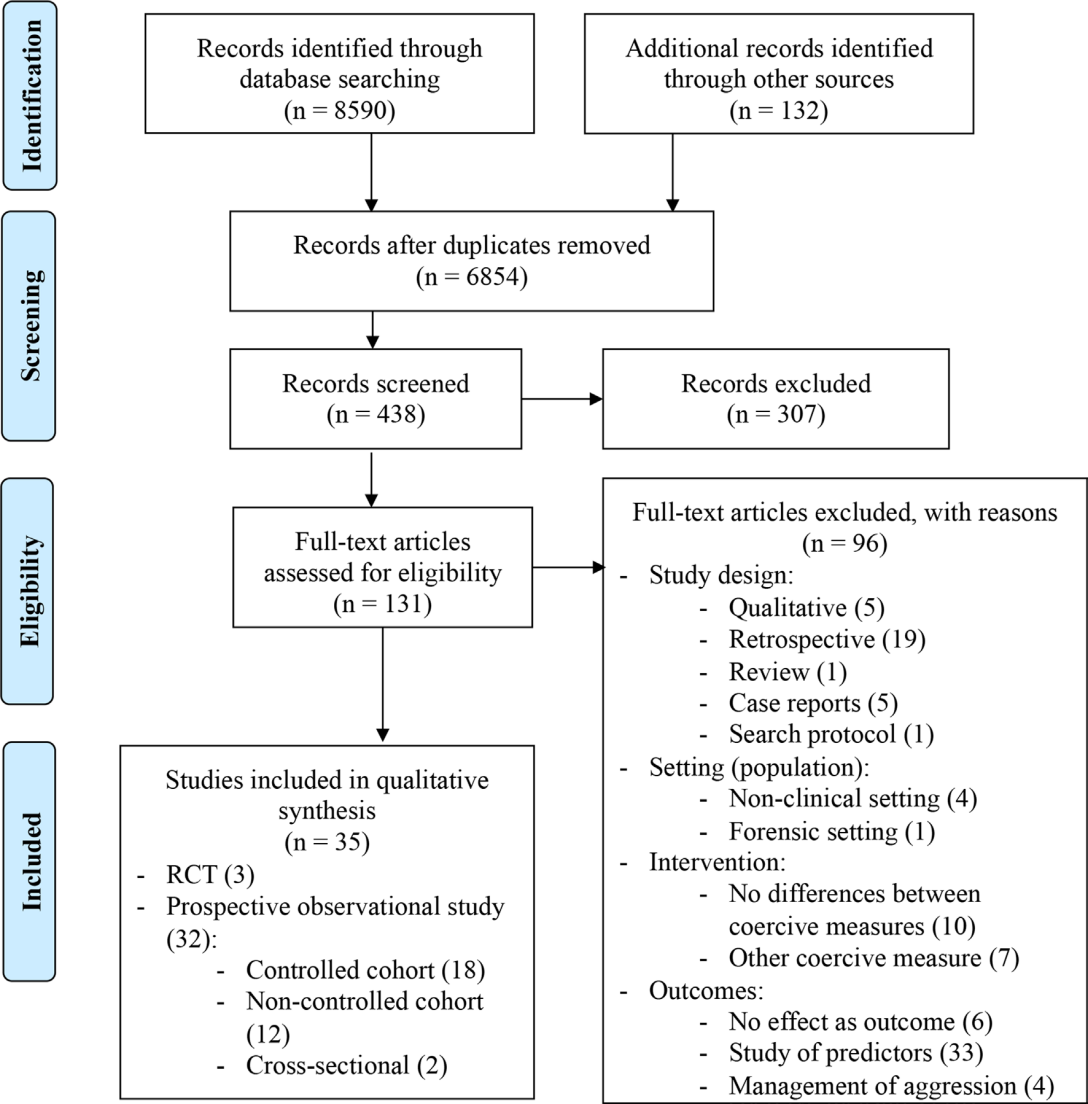
Prisma-Flow Diagram (43).

Search strategies are detailed in **Supplementary Table 1**. We designed comprehensive searches for the two main databases (MEDLINE *via* Pubmed and Embase), described in **Supplementary Table 1**. For less exhaustive databases, we employed the following keywords: (coercion OR restraint OR seclusion) AND (psychiatric OR psychiatry OR mental health) AND (effect OR safety OR harm OR efficiency OR efficacy OR beneficence OR risk OR mortality OR quality of life OR effectiveness). References of selected studies and reviews on seclusion and/or restraint were screened and referred to as “other sources” in **Figure 1** (19, 25, 27–28, 29, 38–40).

### Data Sources, Study Selection, and Data Extraction

#### Data Management, Including Time Frame

The systematic literature search was conducted from the first available article to December 8, 2018. Databases searched have been updated since this date. Duplicates were removed before screening titles with EndNote^™^ X8.2.

#### Study Selection Processes

Two authors independently screened titles and abstracts for study selection. Disagreements were resolved by consensus. The eligibility of retrieved full-text articles was discussed with a third author.

#### Data Collection Processes

Data were extracted from selected articles using specified fields: author, year of publication, location, design and sample, studied interventions, explored outcomes, results, and risk of bias. When available, we reported quantitative results (percentages).

#### Data Analysis

A qualitative analysis of included studies was performed. Due to the heterogeneity of the outcomes reported, a quantitative analysis was not possible. The quality of evidence and risk of bias were systematically assessed for individual studies using the revised Cochrane Risk of Bias Tool (31) for RCTs and the U.S. Preventive Services Task Force (USPSTF) tool for observational studies (41, 42). Several potential sources of bias were evaluated depending on study design. For RCTs, assessed sources of bias were sequence generation, allocation concealment, blinding of participants, personnel and outcome assessors, selective outcome reporting, and other sources of bias. As described in the Cochrane Collaboration Handbook (31), RCTs can then be assigned to different categories of risk (low, moderate, or high). For observational studies, analyzed sources of bias were selection bias (assembly and maintenance of comparable groups), quality bias (adequacy of measurements and potential confounders with either restriction or measurement for adjustment in the analysis), information bias (loss to follow-up and definitions of interventions and outcomes), and selective outcome reporting. Meta-biases (selection and publication, information, and analysis bias) were evaluated following Institute of Medicine guidelines (42).

## Results

### Study Selection and Characteristics

Applying the search strategy described above, we retrieved 8,590 articles from all databases (**Figure 1**), and 6,854 remained after removing duplicates. There were 438 articles eligible for abstract review, and 131 were eligible for full-text reading. Out of these 131, thirty-five studies were included in the qualitative analysis. In all, 96 articles were not related to the research question or did not meet inclusion criteria and were excluded. In terms of PICOS, exclusion criteria addressed the study design: 5 studies were qualitative, 19 were retrospective, 5 were case reports, 1 was a search protocol, and 1 was a review; the population/setting: one study took place in a forensic ward and four in a non-clinical setting; the interventions: 10 made no differences between types of coercive measures, and 7 did not study seclusion or restraint; and finally, the outcomes: 6 did not study effects of coercion but other outcomes, 33 studied predictive factors, and 4 studied aggression and its management but not the effects of seclusion or restraint (**Figure 1**). The characteristics of selected studies are arranged by study design, explored outcome, and comparator to the main intervention (**Table 2**). Three studies were randomized controlled trials, and 32 had a prospective observational design (30 cohorts and two cross-sectional studies). Four studies compared secluded versus restrained patients (32, 44, 56, 62). Two studies compared seclusion and restraint (without distinction) versus non-exposure (48, 58), and four studies compared these measures to other coercive measures (34, 36, 47, 63). Nine studies compared secluded versus non-secluded patients. Guzmán-Parra et al. compared restraint versus forced medication (57), and Wallsten et al. compared restraint versus non-exposure (37). Two additional studies provided data on secluded or restrained patients (without distinction) (66, 67), two examined only restrained patients (35, 55), and 10 studies examined only secluded patients. Diagnoses could differ between studies, but most diagnoses were psychotic disorders (ranging from 26.8% to 82.3%), followed by affective disorders (in particular mania) (varying from 12% to 53.6%), substance use (ranging from 4.9% to 32%), and personality disorders (varying from 1.9% to 11%) (**Table 2**). Two articles did not give diagnostic information (45, 65). Two studies selected patients based on the diagnosis of schizophrenic or schizoaffective disorder (52, 61). Symptom intensity was evaluated with the Positive and Negative Syndrome Scale (PANSS) (32, 33); the Brief Psychiatric Rating Scale (BPRS) (34–37), and/or the Beck Depression Inventory II (BDI-II) (36).

**Table 2:** Characteristics of included studies.

Article	Design and methods	Intervention vs comparator	Explored outcomes	Results and conclusions
Huf et al. 2012, Brazil (44)	- Unblinded RCT, 14-day follow-up - 105 agitated psychotic patients (54 secluded, 51 restrained) - Dg (restrained vs secluded): 82.3 vs 77.8% psychosis (SD or mania), 5.9 vs 11.1% psychological agitations, 11.8 vs 11.1% SU	Seclusion vs restraint	- Effectiveness - Adverse events - Subjective perception	- Negative effect - 2/3 secluded patients fully managed with seclusion, 1/3 changed to restraint - No significant difference between groups in effects, adverse events, or patients’ satisfaction - Ccl: Suggestion to begin with seclusion that seems not to harm or prolong coercion
Bergk et al. 2011, Germany (32)	- Unblinded RCT - 102 patients (12 randomized/48 nonrandomized secluded, 14 randomized/28 nonrandomized restrained Semi-structured interview - Dg (randomized vs nonrandomized secluded/randomized vs nonrandomized restrained): 50 vs 71/86 vs 50% SD, 50 vs 8/14 vs 25% AD, 0 vs 21/0 vs 25% PD	Seclusion vs restraint	- Symptom intensity - Levels of needed medication - Adverse events - Subjective perception	- Negative effect - No significant differences for adverse events and subjective experience - Levels of medication and aggressive symptoms are only significantly lower for nonrandomized secluded patients - Ccl: Clinical decisions should take patients’ preferences into account. RCTs on coercion are feasible
Vaaler et al. 2005, Norway (33)	- Non-inferiority RCT - 25 secluded patients in a traditional manner; 31 in a redecorated room - Dg (new interior vs traditional interior): 51.6 vs 24% SD, 16.1 vs 28% AD, 16.1 vs 24% SU, 6.5 vs 4% OD and 9.7 vs 2% O	Seclusion	- Ward environment - Length of stay - Symptom intensity - Subjective perception	- Negative and beneficial effects - No significant differences between groups - Ccl: No negative effects of a refurnished room on seclusion efficacy
Cashin 1996, Australia (45)	- Prospective quasi-experimental study - 53 involuntary admissions (27 secluded patients, 26 non-secluded) - No diagnostic information but no significant difference between groups	Seclusion vs non-exposure	Time to emergency resolution Levels of needed medication	Beneficial effect No significant differences between groups Ccl: Seclusion may be the most effective choice in some circumstances
Hafner et al. 1989, Australia (46)	- 38-weeks multi-center prospective study - 30 secluded and 60 non-secluded patients - Dg (secluded, no difference between groups): 46.3 (vs 23% non-secluded) SD, 12.2% BPD, manic state, 12.2% MDD, 9.8% OD, 7.3% PD, 9.8% SU, 2.4% BRP	Seclusion vs non-exposure	- Levels of needed medication - Length of stay - Readmission rate	- Negative and beneficial effects - 25% more neuroleptic medication for secluded patients, suggesting that seclusion did not permit to reduce the levels of medication required to manage psychiatric agitation - Less medication for non-secluded patients, suggesting that secluding agitated patients may reduce the unit level of dangerousness - No differences in length of stay or readmission rate, suggesting no adverse effect of seclusion
Georgieva et al. 2012, Netherlands (47)	- 3-year prospective study - 125 coerced patients (62 secluded, 18 forced medicated, 34 secluded and forced medicated, 11 secluded and restrained) - Structured questionnaires - Dg (secluded/involuntary treated/secluded and treated/secluded and restrained): 27/39/53/60% SD, 34/33/38/10% AD, 9/33/9/0% PD, 32/28/13/30% SU, 5/0/6/0% PTSD	Seclusion and restraint vs other coercive measures	- Effectiveness - Adverse events - PTSD - Subjective perception	- Negative effect - Combined seclusion and restraint with higher psychological and physical burden than seclusion alone or seclusion and forced treatment - No significant difference in effectiveness - Ccl: Forced medication seems better tolerated. Seclusion and/or restraint could give revival of previous traumatism or PTSD
Soininen et al. 2013b, Finland (48)	- 1-year prospective study - 36 secluded or restrained (no distinction) patients, 228 non-exposed - Structured questionnaire - Dg (secluded vs non-secluded): 54 vs 33% SD, 31 vs 49% AD, 14 vs 18% O	Seclusion and restraint vs non-exposure	Quality of life	- Beneficial effect - Exposed patients reported a better subjective quality of life at discharge compared to non-exposed patients - Ccl: Either seclusion and restraint had only short-term negative influence on quality of life, or the observed association may not be causal
McLaughlin et al. 2016, 10 European countries (34)	- Multi-center prospective study (EUNOMIA project) - 2,030 involuntary admissions, 770 with one or more coercive measures (84 secluded, 439 restrained, 556 forced medication). - 1,353 interviews - Dg (coerced vs non coerced): 68 vs 60% SD	Seclusion and restraint vs other coercive measures	Length of stay	- Negative and beneficial effects - At 3 months, 843 involuntary admitted patients approved and 506 (37.4%) disapproved their previous admission. Forced medication was the only significant measure associated with admission disapproval - Seclusion and restraint were associated with increased length of stay (in multivariate analysis, only seclusion remains significant). Secluded patients’ symptom intensity did not fully explain the observed increase
Soloff et Turner 1981, US (49)	- 8-month prospective study - 59 secluded patients, 159 non-secluded - Structured questionnaire - Dg (secluded vs non-secluded): 42.4 vs 40.9% SD, 5.1 vs 1.9% BPD, 11.9 vs 11.3% other AD, 6.8 vs 4.4% OD, 8.5 vs 12.6% PD, 0 vs 11.3% neurosis, 23.7 vs 17.6% O (SU and MR)	Seclusion vs non-exposure	Length of stay	- Beneficial effect - Length of stay associated with incidence of seclusion, but no influence of chronicity and legal status at admission - Initial postulate: Seclusion as therapeutic and control function for patient and ward milieu
Schwab et Lahmeyer 1979, US (50)	- 6-month prospective study - 52 secluded patients, 90 non-secluded - Dg (secluded vs non secluded): 29 vs 29% SD, 19 vs 7% BPD, manic state, 14 vs 14% psychotic MDD, 14 vs 32% neurosis, 8 vs 3% SU, 6 vs 3% PD, 10 vs 12% O	Seclusion vs non-exposure	Length of stay	Negative effect Increased length of stay for secluded patients
Mattson et Sacks 1978, US (51)	- 1-year prospective study - 63 secluded patients, 160 non-secluded - Dg (secluded vs non secluded): 63 vs 38% SD, 17 vs 4% BPD, manic state, 10 vs 14% PD, 10 vs 44% O	Seclusion vs non-exposure	Length of stay	- Negative effect - Increased length of stay for secluded patients - Effect no longer significant when focusing on patients less than 20 years of age
Hammill et al. 1989, US (52)	- Prospective study - 100 patients (26 secluded, 74 non-secluded) with SD or SAD - Semi-structured interview	Seclusion vs non-exposure	- Length of stay - Subjective perception	- Negative and beneficial effects - Increased length of stay for secluded patients - 13/17 secluded patients evaluated seclusion as necessary
Plutchik et al. 1978, US (53)	- 2 prospective studies - 1st: descriptive (118 secluded patients, 118 randomly assessed non-secluded) - 2nd: qualitative (30 secluded and 25 non-secluded patients) - Structured interview - Dg (secluded vs non secluded): 64 vs 45.8% SD, 2.5 vs 0% BPD, manic state, 3.4 vs 8.5% psychotic MDD, 10.2 vs 13.6% depressive neurosis, 0.8 vs 5.1% SU, 6.8 vs 13.6% PD, 5.9 vs 8.5% adjustment reactions, 3.4 vs 5.1% OD, 2.5 vs 0% MR	Seclusion vs non-exposure	- Length of stay - Subjective perception	- Negative and beneficial effects - 1st study: Increased length of stay for secluded patients - 2nd study: 40% secluded patients rated seclusion as not helpful. 60% reported feeling better after seclusion
Mann et al. 1993, US (54)	- 6-month prospective study - 50 secluded patients - Structured questionnaire - Dg: 24% MDD, 10% dysthymic disorders, 30% BPD, 2% SAD, 16% SD, 6% BRP, 8% SU, 4% none	Seclusion	- Length of stay - Subjective perception	- Negative and beneficial effects - Seclusion safe and secure (67%) - Feelings of constant attention and care from staff (45%) - Increased length of stay for secluded patients (compared to general unit mean)
Ishida et al. 2014, Japan (55)	- Prospective study - 190 restrained patients - Dg: 3.9% OD, 9.9% SU, 63.5% SD, 14.9% AD, 1.1% somatoform disorders, 6.6% PD	Mechanical restraint	Adverse effects	- Negative effect - D-dimer augmentation for 72 restrained patients with prophylaxis. - US Doppler of lower extremities showed asymptomatic DVT in 21 patients (11.6%) - Incidence of DVT associated with excessive sedation, longer duration of restraint, lower antipsychotic dosage - Ccl: Probable underestimation of DVT in routine use of restraint
Steinert et al. 2013, Germany (56)	- Cross-sectional study, 1-year follow-up after Bergk et al. 2011 - 60 of 102 (59%) previous patients (31 secluded, 29 restrained) - Dgs: 63% SD, 23% BPD, 14% O	-Seclusion vs restraint	- PTSD - Subjective perception	- Negative and beneficial effects - Seclusion reported as less restrictive - 1 secluded and 2 restrained patients with symptoms fulfilling PTSD diagnosis - Ccl: The lower than expected incidence of PTSD may be due to natural resolution of symptoms or to the interviews conducted with the patients, which could have helped prevent PTSD
Guzmán-Parra et al. 2018, Spain (57)	- 2-year prospective study - 111 coerced patients (32 restrained, 41 forced medicated, 38 forced medicated and restrained) - Dg (restrained vs involuntary treated vs combined): 4.9 vs 9.4 vs 10.5% SU, 58.5 vs 50 vs 68.4% SD, 22 vs 28.1 vs 18.4% AD, 2.4 vs 3.1 vs 0% anxiety disorders, 7.3 vs 6.3 vs 0% PD, 4.9 vs 3.1 vs 2.6% O	Mechanical restraint vs forced medication	- PTSD - Subjective perception	- Negative effect - Higher perceived coercion with restraint (compared to forced medication). - Higher post-traumatic stress with forced medication - Combined forced medication and restraint associated with higher coercion perception and less treatment satisfaction (than restraint or forced medication alone)
Steinert et al. 2007, Germany (58)	- Prospective study - 117 involuntary admissions with history of seclusion or restraint, 18 secluded or restrained (no distinction) patients at present admission - Structured questionnaires - Dg: 79.5% SD 8.5% other psychotic disorders, 12% SAD	Seclusion and restraint vs non-exposure	- Influence of history of life-threatening events on traumatic effects of intervention	- Negative effect - Bidirectional association of history of seclusion or restraint with life-threatening traumatic events. - Exposure to past traumatic events enhances the risk of revictimization and revival of previous traumatism during inpatient treatment - Ccl: Coercive measures may cause re-experienced traumatism
Wallsten et al. 2008, Sweden (37)	- 2-year prospective study - 115 patients (19 reported mechanically restrained but 8 false positives; 98 reported non-restrained but 4 false negatives); 15 truly restrained - Structured interview - Dg (true positives/true negatives/false positives/false negatives): 46/52/38/25% SD, 36/9/63/25% AD, 18/19/-/50% O	Mechanical restraint vs non-exposure	- Discrepancy between objective and reported coercion - Subjective perception	- Negative effect - 42% false positive and 4% false negative reports of restraint. - Causes are not clear [communication problem, memories failures (or false memories), or emotional traumatic reactivation] - Ccl: Subjective quality of reports of past traumatic events
Whitecross et al. 2013, Australia (59)	- 9-month prospective study - 31 secluded patients - Dg: 51.6% SD, 32.3% SAD, 16.1% O	Seclusion	PTSD	- Negative effect - 47% probable PTSD (IER-S >33) after seclusion
Fugger et al. 2015, Austria (35)	- 18-month prospective study - 47 mechanically restrained patients - Dg: 23.4% OD, 12.8% SU, 19.1% paranoid SD, 8.5% catatonic SD, 4.2% SAD, manic state, 14.9% BPD, manic episode, 2.1% BPD, mixed episode, 2.1% recurrent MDD, 6.4% anorexia, 6.4% PD	Mechanical restraint after intervention	- PTSD - Subjective perception	- Negative and beneficial effects - 50% high perceived coercion and 25% probable PTSD - Less memory event, more feeling of being healthy and more acceptance of restraint than rated by physicians
Palazzolo 2004, France (60)	- 6-month prospective study - 67 secluded patients - Semi-structured interview - Dg: 32.8% SD, 28.4% BPD, 14.9% BRP, 10.4% SAD, 5.9% anorexia, 4.6% somatoform disorders, 3% antisocial PD	Seclusion	- Hallucinations - Subjective perception	- Negative and beneficial effects - Anger was the most frequent reported emotion - 31% reported hallucinatory experience - 67% reported anxiety - 8% reported feeling better, and 8% the necessity of continuing treatment
Kennedy et al. 1994, US (61)	- Prospective study - 25 secluded patients with SD or SAD - Semi-Structured interview	Seclusion	- Hallucinations - Subjective perception	- Negative and beneficial effects - For 48%, seclusion was not helpful - 52% reported hallucinations during seclusion - 70% who experienced hallucinations during seclusion were hallucinating before seclusion but proportional increase of hallucinations during seclusion was not significant - Hallucinating patients had longer (but not significantly) seclusion stay, more therapeutic interaction (nurse-patient relationship) and levels of needed medication
Sagduyu et al. 1995, US (62)	- Prospective study - 25 secluded and 25 restrained patients - Semi-structured interview - 76% restrained and 80% secluded patients had a SD	Seclusion vs Restraint	Subjective perception	- Negative and beneficial effects - 40% secluded and 20% restrained with positive evaluation - 71% secluded and 89% restrained remembered past traumatic experiences - 73% secluded and 81% restrained reported negative feelings
Krieger et al. 2018, Germany (36)	- 18-month prospective study, - 213 involuntary admitted patients (78 mechanically restrained, 32 secluded, 30 forced medicated, 20 video monitored) - 51 voluntarily admitted patients in a closed ward, - Structured interview - Dg (coerced vs control groups): 71.1 vs 51% SD, 10 vs 21.6% SU, 12.8 vs 19.6% AD, 3.3 vs 7.8% PD, 33.6 vs 45.1% of comorbidities with SU	Seclusion and restraint vs other coercive measures	Subjective perception	- Negative and beneficial effects - Negative emotions associated with seclusion or restraint - Increasing understanding of use of seclusion or restraint during hospitalization - Seclusion preferred among all coercive measures, while restraint less accepted than the other measures
Gowda et al. 2018, India (63)	- Prospective study - 200 patients (40 mechanically or manually restrained, 36 secluded, 116 chemical restrained, 64 involuntarily treated, 29 ECT) - Dg: 48% SD, 43.5% AD, 18.5% O, 48.5% comorbidities with SU	Seclusion and restraint vs other coercive measures	Subjective perception at admission and discharge	Negative effect Physical restraint associated with a greater perception of coercion, followed by involuntary treatment, chemical restraint, seclusion and finally ECT
Sorgaard 2004, Norway (64)	- 17-week prospective interventional study - 190 admissions (16% secluded, 160 non-secluded) - Standardized questionnaires - Dg (baseline vs project phase): 26.8 vs 28.6% SD, 53.6 vs 41.2% AD, 3.6 vs 5.0% PD, 8.9 vs 11.8% SU, 7.1 vs 13.6% O	Seclusion vs non-exposure	- Adverse events - Subjective perception	- Negative effect - Seclusion as principal factor associated with perceived coercion (compared to age, sex, forced medication, or length of stay)
Martinez et al. 1999 (65)	- Cross-sectional study - 69 patients (53 secluded, 16 non-secluded) - Semi-structured interview - No diagnostic information	Seclusion vs non-exposure	Subjective perception	- Negative and beneficial effects - Negative perception of seclusion (62% overuse, 76.5% punishment) - 56.2% reported seclusion as needed
Larue et al. 2013, Canada (66)	- 1-year prospective study - 50 secluded or restrained (no distinction) patients - Semi-structured interview - Dg: 66% SD, 30% AD, 2% PD, 2% anxious disorders	Seclusion and restraint	Subjective perception	- Beneficial effect - 52% agreed with improved behavior after seclusion
Soininen et al. 2013a, Finland (67)	- 18-month multi-center prospective study - 90 secluded or restrained patients (no distinction) - Structured questionnaire - Dg: 12% SU, 60% SD, 20% AD, 6% PD	Seclusion and restraint	Subjective perception after intervention	- Negative effect - Deny necessity and beneficence of seclusion or restraint - Dissatisfaction - Not enough dialogue
Keski-Valkama et al. 2010, Finland (68)	- 1-year prospective study - 38 secluded patients in general vs 68 in forensic wards - Structured interview - Dg in general wards: 71.1% SD, 10.5% SU, 15.8% AD, 2.6% O	Seclusion	Subjective perception	- Negative and beneficial effects - Mostly negative feelings, loneliness - Need for interaction - Seclusion as necessary - 54% secluded patients perceived seclusion as a punishment
Stolker et al. 2006, Netherlands (69)	- 18-month prospective study - 78 secluded patients - Structured interview - Dg: 67% SD, 11% BPD, 11% cluster B PD	Seclusion	- Ward environment - Subjective perception	- Negative and beneficial effects - Staying in multi-bed rooms prior to seclusion associated with less negative views of seclusion
Richardson et al. 1987, US (70)	- 1-year prospective study - 52 secluded patients - Semi-structured interview - Dg: 36.5% SD, 28.8% SAD, 19.2% AD, 9.6% atypical psychosis, 1.9% borderline PD, 1.9% organic hallucinosis, 1.9% dementia	Seclusion	Subjective perception	- Negative and beneficial effects - 31% patients reported anger, 58% felt punished - 50% reported seclusion as protection, 48% as necessary - 37% reported hallucinatory experience - 20/52 reported improvement after seclusion, 8/52 deterioration
Binder et McCoy 1983, US (71)	- 8-month prospective study - 27 secluded patients - Semi-structured interview - Dg: 45.8% SD, 33.3% AD, 8.3% SAD, 8.3% antisocial PD, 4.2% acute paranoid BRP	Seclusion	Subjective perception	- Negative and beneficial effects - 4 patients rated seclusion as therapeutic, 12 as necessary - 11 rated beneficial aspects (7 hypostimulation) - 18 negative emotions - For 14, seclusion had no effect, 3 beneficial effect, 2 negative effect, 5 first negative effect changed to beneficial effect
Tooke et Brown 1992, US (72)	- 11-week prospective study - 19 secluded patients (11 locked rooms, 8 secluded area) - Structured questionnaire - Dg: 47.3% SD, 26.3% MDD or suicidal ideations	Seclusion	Subjective perception	- Negative effect - 73% secluded patients (in locked rooms) felt punished - Strong negative feelings

vs, versus; RCT, randomized controlled trial; Dg, diagnoses; Ccl, conclusions; SD, schizophrenic disorders; AD, affective disorders; PD, personality disorders; SAD, schizoaffective disorder; BRP, brief reactive psychosis; SU, substance use; O, others; BPD, bipolar disorders; MDD, major depressive disorder; OD, organic disorders; MR, mental retardation; PTSD, post-traumatic stress disorder; EUNOMIA, European Evaluation of Coercion in Psychiatry and Harmonization of Best Clinical Practice; US, ultrasound; DVT, deep vein thrombosis; IES-R, Impact of Event Scale-Revised; ECT, Electro-convulsive therapy.

### Synthesized Findings

Overall, evidence for negative effects have consistently been found across studies: PTSD (47, 57–59), medication need (46), increased length of stay (34, 50–54), deep vein thrombosis (DVT) (55). One study suggested a beneficial effect on quality of life (48). Drawing clear conclusions on beneficial effects of seclusion and restraint were not allowed. Effects of these measures included various outcomes (**Table 1**): two studies explored objective effectiveness of seclusion and restraint (45, 46), four examined beneficial effects, adverse effects, and subjective perception (32, 33, 44, 47), one examined adverse effects (55), one examined quality of life after seclusion or restraint (without distinction) (48), four examined the influence of seclusion or restraint on length of stay (34, 49–51), three examined length of stay and subjective perception (52–54), one examined the incidence of PTSD after seclusion or restraint (58), four examined incidence of PTSD and subjective perception (35, 56, 57, 59), two examined reported hallucinatory experiences during seclusion and subjective perception (60, 61) and 13 examined subjective perception of seclusion or restraint. Fourteen studies reported negative effects of seclusion and restraint, four reported beneficial effects, and 17 reported negative and beneficial effects. Results for these heterogeneous outcomes strongly diverge across studies. Some of them have struggles in achieving definitive conclusions, namely, regarding subjective outcomes. Below, we detail results for each explored outcome by study design and comparator to the main intervention.

#### Objective Effectiveness

In Bergk et al. and Huf et al., two RCTs comparing seclusion versus restraint, the two interventions had similar effectiveness in terms of level of needed medication (32, 44), intensity of aggressive symptoms, and safety during and after interventions (32). Level of needed medication and aggressive symptoms were significantly lower for the secluded, nonrandomized group compared to the secluded, randomized or restrained (randomized or nonrandomized) groups (32).

Georgieva et al., a prospective study, compared effectiveness (evaluated through global functioning and reduced aggression) between different coercive measures: seclusion or forced medication alone or seclusion combined with forced medication or restraint (47). Seclusion combined with restraint was not more effective than seclusion or forced medication alone or combined (47). In McLaughlin et al., a prospective multi-center study, length of stay was increased for seclusion, restraint, or forced medication compared to non-exposure (to each evaluated coercive measure, but only seclusion remained significant in multivariate analysis) (34). In Soininen et al., secluded or restrained patients (without distinction) reported better subjective quality of life at discharge compared to non-exposed patients (48). The authors concluded that, on the one hand, seclusion and restraint had only little or short-term negative influence on quality of life, and on the other hand, the observed association may not be causal. For them, variations in diagnosis between groups of patients could explain the observed differences (majority of mood disorders in non-exposed versus schizophrenia for exposed patients). Mood disorders are indeed associated with lower subjective quality of life in the literature (48).

In a prospective quasi-experimental study comparing seclusion and non-exposure, Cashin identified no significant differences for level of needed medication or resolution time for emergencies (45). Hafner et al. compared two units prospectively, one using seclusion and the other not using seclusion (46). Seclusion was compared to non-exposure in the same unit on one hand and to the other unit on the other. Secluded patients needed 25% more medication than those agitated but non-secluded in the unit not using seclusion. The authors concluded that seclusion was not sufficient to treat agitation as more medication was needed. On the other hand, in the unit using seclusion, non-secluded patients needed less medication than those in the unit not using seclusion, suggesting that seclusion could reduce the dangerousness of the ward (46). Several prospective studies found an increased length of stay for secluded versus non-exposed patients (49–54). However, in Mattson and Sacks, this effect was not significant when focusing on patients less than 20 years of age (51).

In Vaaler et al., an RCT comparing seclusion in furnished and unfurnished rooms, no negative influence of furniture was found on effectiveness in terms of length of stay or symptom intensity (33).

#### Adverse Effects

In Bergk et al. and Huf et al., the two RCTs comparing seclusion versus restraint, no significant differences between the two interventions were found for adverse events during or after the intervention in terms of agitation, suicide attempt or self-harm, fracture, revival of previous traumatism, death, hypertension, or physical pain (32, 44). Although 40% of patients were stated to be at risk for PTSD after seclusion or restraint in Bergk et al.’s RCT (32), only one secluded and two restrained patients had symptoms fulfilling PTSD diagnosis at a 1-year follow-up (56). These authors concluded that this lower than expected incidence of PTSD may be due to natural resolution of symptoms or to the interviews conducted with the patients, which could have helped prevent PTSD (56).

When comparing seclusion and forced medication alone or combined, or seclusion with restraint, Georgieva et al. reported more adverse events for seclusion combined with restraint than for seclusion or forced medication alone or combined (47). In a prospective study involving involuntarily admitted patients, Steinert et al. found a bidirectional association between history of seclusion or restraint (without distinction) and life-threatening traumatic events (58). Thus, the authors concluded that, on the one hand, exposure to past traumatic events could enhance risk of victimization and revival of previous traumatism during inpatient treatment, and on the other hand, seclusion or restraint may cause re-experienced traumatism.

In Guzmán-Parra et al., comparing restraint to forced medication, more traumatic experiences were reported after restraint (57).

In three prospective studies, 31% to 52% of secluded patients reported hallucinatory experiences (60, 61, 70). In the Kennedy study, 70% of the 52% reported hallucinations were present before seclusion and the increased intensity during the intervention was not sufficient to conclude that seclusion may cause the hallucinations (61). Hallucinating patients had longer seclusion time and more therapeutic interaction and medication than non-hallucinating patients (61).

In Ishida et al., a prospective study involving mechanically restrained patients receiving prophylaxis, Doppler ultrasound of lower extremities showed 11.6% incidence of asymptomatic deep vein thrombosis. The authors concluded, therefore, that there was probably underestimation of deep vein thrombosis in routine use of restraint (55). In an observational study, Fugger et al. found a 25% incidence of PTSD after mechanical restraint (29% at hospital discharge and 22% at 4 weeks after discharge) (35), while Whitecross et al. found a 47% incidence of PTSD after seclusion (59).

#### Subjective Outcomes

In Bergk et al. and Huf et al., patients’ preferences between seclusion and restraint were not significantly different (32, 44). After 1-year follow-up in Bergk et al.’s RCT (32), 58% secluded or restrained patients reported positive emotions, but mechanical restraint was assessed more negatively than seclusion (56). In Sagduyu et al., a prospective study comparing seclusion versus restraint, 40% secluded and 20% restrained patients evaluated the intervention as beneficial (62). Additionally, 71% secluded and 89% restrained patients remembered past experiences (confinement or physical abuse), and 73% secluded and 81% restrained patients reported negative feelings (62).

In Georgieva et al., seclusion combined with restraint was associated with higher perceived coercion than seclusion or forced medication alone or combined (47). In Krieger et al., comparing various coercive measures (involuntary admission combined with seclusion, mechanical or manual restraint, forced medication or video monitoring) to non-exposure (voluntary admission in a closed ward without other coercive measure), more negative emotions were related to seclusion or restraint (36). Patients’ understanding of use of seclusion or restraint increased during hospitalization, and seclusion was preferred among all coercive measures, while restraint was less accepted than the other measures (36). In Gowda et al., another prospective study comparing perceptions of coercion from seclusion, physical (mechanical or manual) or chemical restraint, involuntary treatment, and electroconvulsive therapy at admission and discharge, physical restraint was associated with a greater perception of coercion, followed by involuntary treatment, chemical restraint, seclusion, and finally electroconvulsive therapy (63).

In Sorgaard, an interventional study, seclusion was the main factor associated with perceived coercion compared to age, sex, forced medication, or length of stay (64). In Martinez et al., a cross-sectional study, seclusion was rated as needed in 56.2% of cases but was mainly associated with negative perception (62% overuse and 76.5% punishment) (65).

In Guzmán-Parra et al., restraint was associated with a greater perception of coercion than forced medication (57). In Wallsten et al., a prospective study evaluating adequate patient reports of mechanical restraint, four restrained patients reported not having been restrained, while eight non-restrained patients reported having been restrained. The cause of these eight false positive and four false negative reports was not clear, as it could be due to communication problems, memory failures (false memories for false positive) or emotional traumatic reactivation (37). In this study, the authors raised the question of the subjectivity of patients’ self-reports of coercion.

After seclusion or restraint (without distinction), patients reported a feeling of clinical improvement (66), as well as dissatisfaction, denial of necessity or beneficence, and insufficiency of dialogue with staff (67).

In Stolker et al., a prospective study evaluating the influence of the ward environment on perceptions of seclusion among secluded patients, perceived coercion was lower in cases of previous stays in multi-bed rooms compared to stays in single rooms (69). The authors concluded that the subjective effect of seclusion on patients could depend on the ward environment. In several prospective studies, seclusion was positively evaluated as safe and secure (54, 70) and slightly necessary (52, 70, 71). In three studies, patients reported feeling better after seclusion (53, 70, 71). In Mann et al., secluded patients reported positive feelings of constant attention and care from staff (54). In Keski-Valkama et al., more therapeutic interaction was demanded by secluded patients (68). Importantly, negative emotions were reported in most studies (70–72). Patients frequently reported seclusion as not helpful (46, 61, 71) or as punishment, ranging from 54% to 73% (68, 70, 72).

In Fugger et al., an observational study comparing patients’ versus physicians’ perceptions of mechanical restraint (during and after intervention), patients’ ratings showed greater perceived coercion but less memory for the event, and greater feelings of being healthy and more acceptance than physicians expected (35).

### Quality Assessment and Risk of Bias

#### RCTs (Revised Cochrane Risk of Bias Tool) (31)

The three included RCTs did not use true allocation. Bergk et al. used an optional randomization (32); Huf et al. could re-allocate some patients when stakeholders evaluated seclusion as not efficient (44). In Vaaler et al., allocation depended on patient number in the unit or previous admittance (on equal headcount) (33). Blinding was not possible due to the characteristics of the measures (seclusion, restraint, or no coercive measure). Selected studies published significant and non-significant results with beneficial and negative outcomes. Missing data, dropout, and reasons for refusing to participate were well documented in the selected studies. We found no identifiable selective outcome reporting in included studies for outcomes, time points, subgroups, or analyses, but few elements were available for detection of potential selective reporting. Registration of trials before study initiation was indeed performed only for Huf et al. (73). For this RCT, predetermined outcomes were identical to the final reported outcomes (44). Intention-to-treat analysis was respected in Huf et al. and Vaaler et al. (33, 44). In Bergk et al., statistical analyses were conducted without six drop-out patients (32). Intention-to-treat analysis was, therefore, not fully respected. Concerning other potential sources of bias, Huf et al. postulated that restraint is more restrictive than seclusion and concluded by suggesting beginning with seclusion (44). The starting hypothesis seems to be identical to the conclusion and, in our opinion, could be a tautology. In Vaaler et al, the authors compared the influence of interior design on seclusion effectiveness measured as symptom intensity and global functioning during and after intervention (33). However, in our view, the study design seems to be unclear. The aim of the study was to detect differences between patients secluded in furnished or unfurnished rooms. Outlined this way, the design seems to be a superiority study. However, the authors stated in the results and discussion that negative effects of furnished rooms on seclusion effectiveness could not be significantly found due to lack of power. Described this way, the null hypothesis that furnished seclusion rooms have a negative impact on seclusion effectiveness could not be rejected. This formulation would correspond to a non-inferiority design. Thus, there seems to be a discrepancy between predetermined study design and results interpretation. This discrepancy creates questions as to the adequacy of the conclusion stating that furnished rooms seem to have no negative effect on seclusion effectiveness. According to the revised Cochrane Risk of Bias Tool (31), and despite following adequate methodological guidelines, the three included RCTs could be assessed as having a high risk of bias, due most notably to non-exhaustive allocation and no ability to use blinding.

#### Prospective Observational Studies (USPSTF tool) (41, 42)

The included prospective studies reported the methods for group constitution and described the group characteristics for assessing comparability. Some studies did not search for confounding factors when an association was found between variables. For example, in several prospective studies, potential confounders were not stated for seclusion or restraint and length of stay (49, 50) or quality of life (48). Some studies had no control group for comparison of results (35, 54, 59, 71, 72). When present, others did not perform subgroup or quantitative analysis for significant differences between groups (62, 65). For some cohort or cross-sectional studies, lack of power and difficulties achieving rejection of the null hypothesis were a problem (45). Selected studies published significant and non-significant results with beneficial and negative outcomes. Missing data, dropout, and reasons for refusing to participate were well documented in the selected studies. We found no identifiable selective outcome reporting in included studies for outcomes, time points, subgroups, or analyses, but few elements were available for detection of potential selective reporting. Registration of trials before study initiation was indeed not performed for included studies, and we could not compare predetermined outcomes with final reported outcomes. Heterogeneity of seclusion and restraint definitions cause difficulties for assessing comparable outcomes. For some studies, seclusion meant open or locked rooms (49, 65), whereas in most studies, seclusion was defined as a locked room from which the patient cannot get out on his own. For other studies, physical restraint could be either mechanical or manual and sometimes both (35, 55, 57). Gowda et al. described the difference between chemical restraint (used during emergencies) and involuntary medication (in case of leverage) (63), while most studies referred to the two categories as involuntary (or forced) treatment. These authors also distinguished between subjective and perceived coercion, whereas most studies used one or the other without differentiation (63).

## Discussion

### Summary of Main Findings

This review synthesizes a wide range of information into an original overview on coercive measures in adult psychiatric patients. Thirty-five articles addressed the search question on beneficial and negative effects of seclusion and restraint in adult psychiatry. The identified literature strongly suggests that seclusion and restraint have deleterious physical or psychological consequences. The incidence of PTSD after seclusion or restraint ranges from 25% to 47%, which is not negligible (35, 59), especially in patients with past traumatic events (58). The main diagnoses associated with the use of seclusion or restraint in the selected articles are schizophrenic, schizoaffective, or bipolar, currently manic disorders. Subjective perception has high interindividual variability and can be positive, with feelings of safety, help (54), clinical improvement (53, 66), or evaluation as necessary (52, 71). However, seclusion and restraint are mostly associated with negative emotions, particularly feelings of punishment and distress (62, 70, 72). Conclusions on protective or therapeutic effects of seclusion and restraint are more difficult to draw. Our results provide little evidence for these outcomes, but further research is clearly necessary. Objective effectiveness of seclusion and restraint seems to be comparable in terms of needed medication, symptom intensity, and adverse effects (32, 34, 44). Compared to non-exposure, they have deleterious physical and psychological consequences, like PTSD, revival of previous traumatism, DVT, increased length of stay, hallucinations, and negative emotions (47, 57, 64). Seclusion seems to be better accepted than other coercive measures, such as forced medication, while restraint seems to be less tolerated (36, 63). A reason could be that seclusion is perceived as a “non-invasive” method (63). Therapeutic interaction seems to influence perceptions of coercion and could help to avoid negative effects when coercive measures are not avoidable (54, 67, 68).

### Overall Assessment of the Quality, Completeness and Applicability of Evidence

We chose to include a broad range of outcomes for effects of seclusion and restraint in adult psychiatry. We found 35 relevant articles with significant or exploitable results, but also a high heterogeneity with respect to the study designs and the explored outcomes. There are only three published RCTs, which point out the challenge of obtaining usable data to prove clinical efficiency and to identify benefits or harms of seclusion and restraint on patients with severe mental disorders. In an era of evidence-based medicine, it shows that daily clinical practices can still be traditional habits, more than therapeutic methods proven to be effective. This finding does not mean that coercive methods are not necessary in certain cases, but in the context of limiting human rights and potential deleterious consequences, the limitations of the evidence base should invite medical and nursing staff to question their practices and to use them with caution and with hindsight when the decision (as a last resort) to use them is made.

Methods for studying effects of coercion in adult psychiatry can be difficult to design. According to the Cochrane Risk of Bias Tool (31) and despite following adequate methodological guidelines, the three included RCTs could be stated as having a high risk of bias (32, 33, 44). However, due to the nature of the topic, it is very difficult to avoid these biases, and the realization and publication of studies are an excellent example of how to deal with the inherent methodological constraints. On the other hand, prospective cohort or cross-sectional studies can have difficulties achieving significant results or statements of causal inference. Lack of power, loss to follow-up, and presence of confounding factors are problems frequently faced in these studies (45, 48). This overview of evidence assessment outlines the difficulties in realizing clinical trials on this subject. Despite the complexities of the issue and the associated challenges, it seems relevant to give an assessment of the current state of the evidence related to seclusion and restraint in adult psychiatric patients, because of the fundamental aspects and the consequences inherent in these measures.

### Comparison With Other Studies and Reviews

The previous review most comparable to ours was published by Sailas and Fenton in 2000, with a last update in 2012 (19). The authors found two RCTs awaiting publication meeting their search criteria, with no evidence of efficacy of seclusion and restraint. Our search criteria applied to the literature published until 2018 allowed us to include 35studies, 3 of which were RCTs. However, even this broader approach could not establish strong evidence of efficacy of seclusion and restraint. Heterogeneity of study designs, studies outcomes, and settings did not allow drawing clear conclusions on beneficial effects of these measures. Overall, this overview shows a very limited progress in establishing efficacy of seclusion and restraint. Thus, it supports the current trend of developing further research and political and juridical regulations, as well as reduction programs targeting these coercive measures.

With our search strategies, we included outcomes like staff attitude or effects of seclusion or restraint on staff, but no article seemed to study this outcome as a direct effect of coercion. Studies on staff perception of seclusion or restraint were found (74, 75) but seemed to evaluate opinion on the topic or risk factors for use of coercive measures more than direct effects of seclusion or restraint. In our opinion, they could not help address the search question.

Another source of limitation when studying coercion is the heterogeneity of definitions. We saw some differences when assessing the risk of bias. In the included literature, we found no difference made between coercion as a measure against the patient’s will and a limitation of freedom of movement, which are two different elements of a coercive measure (1). To achieve a precise and adequate study of coercive measures, it would be important to specify and clarify the implicit dimensions of coercion.

Some studies are frequently cited in articles or reviews. Of those studies, we chose not to include Soliday 1985 and Wadeson and Carpenter 1976 because of thematic analysis of open interviews (76, 77). From our perspective, results did not address our search question regarding objective effect measures. One of the first articles on the topic is Gutheil 1978 that theoretically conceptualized the effects of seclusion as therapeutic (26). This concept has been quoted as a hypothesis in most studies and reviews (49), including Fisher 1994 (2) and Mason 1992 (78), despite the fact that these studies include few evidence-based results.

Some other studies could have been relevant for our search question but did not meet inclusion criteria. For example, Nawaz et al. 2007 conducted an RCT comparing the effectiveness of standard restraint and safety nets but in a geriatric population (79). Generalization of results to the adult psychiatric population seemed difficult, and therefore we did not include the study. Mion et al. 1989 studied physical restraint in a mixed adult, but mainly geriatric, population (80). In our opinion, no conclusion for adult psychiatric patients could be drawn, and we chose not to include the study.

### Implications for Clinical Practice

Concerning clinical practice, Vaaler et al. found no influence of a furnished seclusion room on seclusion effectiveness (33). This study suggested that the settled norm (strictly unfurnished room for seclusion) could be reassessed, and habits could change in ways more agreeable to patients. The reported adverse effects should be taken into account in clinical practice, mainly when deciding the accuracy of using coercive measures on a patient. The incidence of PTSD after seclusion and restraint was not clearly stated and varied widely (from 25% to 47%) (35, 47, 57–59). Steinert et al. and Georgieva et al. found that seclusion and restraint may cause revivals of previous traumatism (47, 58). Difficulties comparing studies are undeniable, but these results show a trend toward potential traumatic experiences after seclusion or restraint. Palazzolo and Kennedy et al. have also reported hallucinatory experiences during seclusion (60, 61), in which occurrence mechanism is not clearly stated. Seclusion and restraint should therefore be used with caution, and staff should closely monitor development of post-traumatic symptoms or hallucinations. Following the hypothesis that the prevalence of DVT is likely to be underestimated under restraint (55), new protocols should be elaborated to prevent these negative effects of restraint when clinical circumstances require its use. Seclusion could be better accepted than restraint (36, 63). Preferred use of seclusion could be a possible change to implement in clinical practice, but it should still be used as a last resort method.

On the other hand, diagnostic variations could be a relevant factor in the use of seclusion or restraint. In the selected studies, patients were more frequently diagnosed with schizophrenic, schizoaffective, or bipolar, currently manic disorders. These results are consistent with the recent literature that reports associations between schizophrenic or organic mental disorders and risk of use of seclusion (81) and coercive measures generally (8, 22, 82). Martin et al. and Miodownik et al. found an augmented risk of seclusion and restraint for patients with schizophrenic disorders (83, 84), and Beghi et al. an increase of restraint for the same diagnoses (27). These associations have implications in clinical practice: psychotic disorders are known to often be chronic and associated with recurrent decompensation. The type of diagnosis could be considered as a moderator and risk factor of long-term use of coercion. Management of schizophrenic and psychotic disorders generally (including mania) should maybe be reassessed in the light of an augmented risk for the use of coercion. Therefore, the need for development of alternatives to coercive measures is a priority, as well as structured research to better understand efficiency of coercive measures and in which context they could be applied. In addition, this research should consider the subjective preference of patients (32).

Subjective perception of seclusion and restraint is mainly associated with negative emotions, like loneliness, helplessness, and feeling of punishment (36, 65, 68). Therapeutic interaction seems to influence perceptions of coercion and could therefore be of importance to help patients coping with these feelings and enhance the therapeutic effect despite the coercive aspect of the measure (85, 86). In several prospective studies, secluded or restrained (without distinction) patients reported feelings of constant attention and care from staff (54), asked for more interaction (68), or evaluated the latter as insufficient (67). Our hypothesis is that interaction with staff permits elaboration of the therapeutic relationship, which mediates treatment outcomes and particularly efficacy (85, 86). This finding suggests that possibilities of patient-staff interactions should be reinforced to develop therapeutic relationships and secondarily improve the effects and subjective perceptions of coercion. The place and meaning of the therapeutic relationship during coercion could therefore also be explored in future research. Helping to develop a secure therapeutic relationship for the patient could be an alternative to the use of seclusion or restraint, as relations as well as measures limiting liberty of movement have a containment function.

### Implications for Research

**Figure 2:**
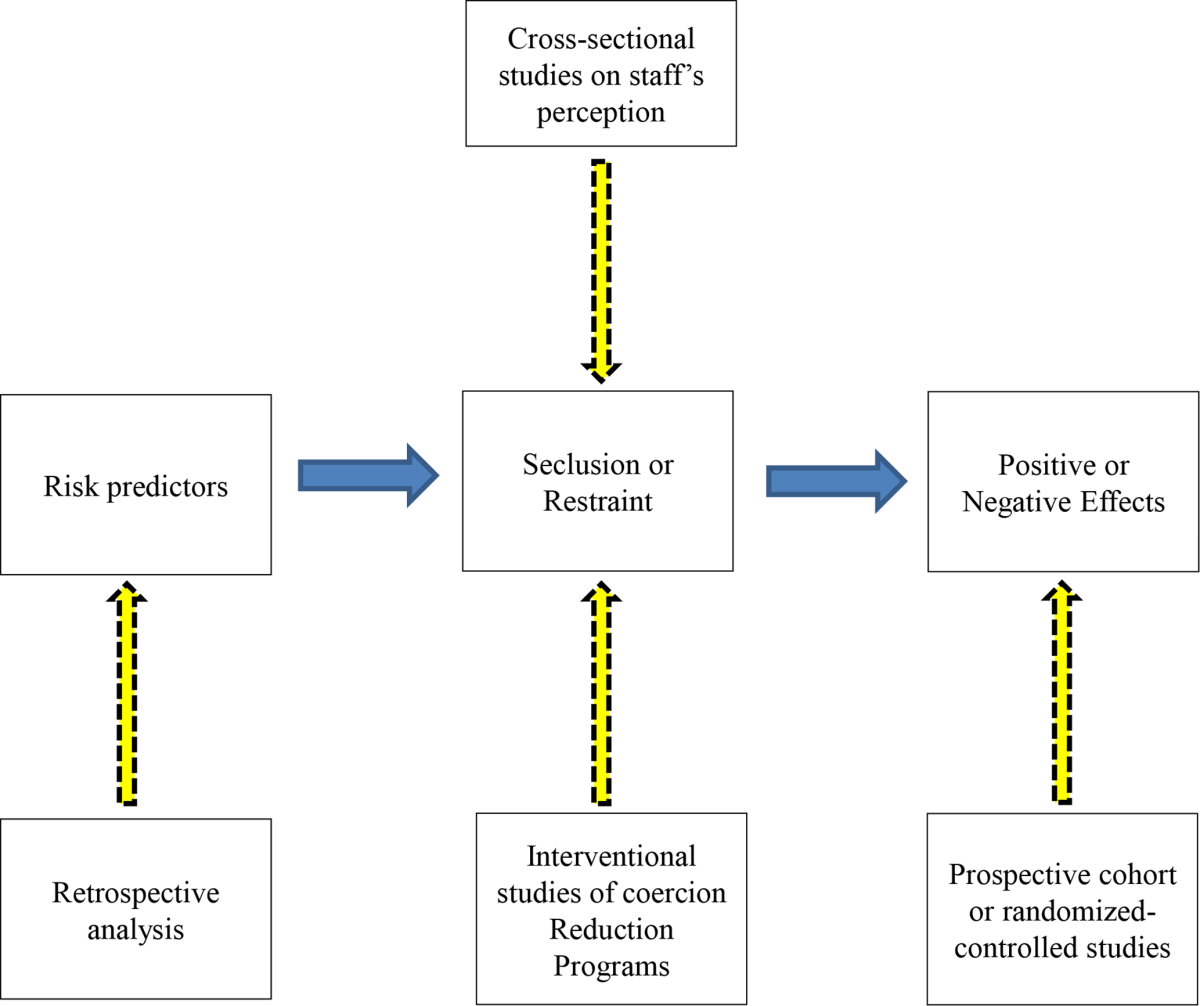
Methodoligical differences in studying risk predictors and effects of seclusion and restraints.

Concerning research, our literature review clearly shows that significant results for effects of seclusion and restraint in adult psychiatry remain difficult to obtain. A reason seems to be the disparity in the topic of comparison as well as the complexity of elaborating study designs for agitated patients. In this review, we focused on prospective studies that allow identification of effects of coercive interventions. However, other approaches have been used in research on coercive measures but may be better suited to assess different outcomes. In the following paragraphs, we propose a brief review of different available methodologies and their appropriateness depending on the outcome of interest (**Figure 2**). In our opinion, specifying these differences is worthwhile for further clinical research in order to adapt the methodology to specific research questions and therefore obtain reliable and valid results. Further research on coercive measures is indeed needed, concerning epidemiology, efficacy, and risk prediction. Its purpose should be the elaboration of seclusion and restraint reduction programs and development of alternatives to their use.

Retrospective methodology is frequently used to determine risk factors and predictors of seclusion and restraint (16, 87). A retrospective methodology may however not be adequate for determining the effects of these measures as it can provide associations among risk factors but does not provide significant and reliable associations among effects of an intervention (**Figure 2**). It seems to us that methodology can greatly influence the significance of the final results.

Another current tendency emphasizes that coercive measures, particularly seclusion and restraint, are last resort methods and, therefore, should be reduced as much as possible. Accordingly, recent articles and reviews focus on reduction programs that have mainly been evaluated with outcomes directly reflecting coercive events themselves, in particular frequency, duration, and other parameters (3, 25). This approach has to be clearly differentiated from our question about the consequences of seclusion and restraint (**Figure 2**). It does not mean that direct study of coercive measures should be discarded as they are still frequently used and are needed as a last resort for difficult situations in clinical practice when no other alternative remains.

In this review, we focused on prospective studies addressing the consequences of seclusion and restraint (**Figure 2**). Scientific evidence of benefit or harm should ideally be investigated with randomized controlled studies (17, 88). However, concerning seclusion and restraint, and coercive measures in general, the feasibility of such studies is controversial (20). Despite an adequate method, the three published RCTs show difficulties in achieving easily interpretable results without high risk of bias. This observation raises the question of whether choosing an RCT design is adequate when studying the effects of coercion. One reason for the lack of data when using an RCT design could be that it is deemed dangerous to conduct a randomized controlled trial of seclusion or restraint. This makes the situation rather similar to that which exists in lethal diseases, for example, surrounding the Ebola outbreaks that raised ethical discussion on feasibility and adequacy of using RCT design when studying efficacy for candidate Ebola vaccines (89). In this ethical discussion, the authors were in favor of using RCT despite methodological difficulties and dangerousness, arguing with four often neglected factors (benefits to non-participants and participants once a trial is over, participants’ prospects before randomization, and the near-inevitable disparity between arms in any randomized controlled trial) (89). When studying seclusion and restraint, the second and third factors seem not to be directly applicable, in particular due to the coercive aspect and implementation against the patient’s will of the measure. These elements open a wide range of questions that would require supplementary reflection and discussion in further researches.

Cross-sectional studies are also often used in research on coercive measures, but investigation of benefits and harms is very limited with this study design. In this context, well-conducted prospective cohort studies seem to be more feasible than RCTs and should produce meaningful results for effects of seclusion or restraint, even though the evidence level will be reduced in comparison to RCTs. This design could allow collection of more useful results and therefore have greater impact on clinical practice changes.

### Strengths and Limitations

The main strength of our review is the broad and systematic search for effects of seclusion and restraint in terms of outcomes and methodology. To our knowledge, this review is the first to synthesize this kind of wide range of information and produce an original overview on the topic, with inclusion of beneficial and negative objective effects and patients’ subjective perception of seclusion and restraint. This review also examined the methodology used for studying coercive measures. To our knowledge, this aspect has thus far not been considered in the literature and could clarify future research perspectives.

Synthesizing assessment of evidence from individual studies highlights some general problems in evaluating the effects of seclusion and restraint in adult psychiatry. Due to heterogeneity of study methods and settings through populations, interventions, comparators, and explored outcomes, synthesizing results and generalization to global conclusions could be at high risk of analysis bias and lead to inaccurate conclusions. For this reason, we tried to compare analogous studies between them and identify some perceptible trends rather than aggregate observations. These trends concern methodology for studying coercive measures on the one hand and effects of seclusion and restraint in adult psychiatry on the other hand.

The width of the conducted search and the selected outcomes are not only strengths but also limitations of our review, as the heterogeneity of the results clearly limits our capacity to draw definitive conclusions. Including objective and subjective outcomes in the same review is a new approach but again renders the integration of findings more difficult. Due to this inclusion of a broad range of outcomes, we had to limit the review to coercive measures limiting freedom of movement (seclusion and restraint). The exclusion of other types of coercion like involuntary admission or treatment is clearly another limitation of this review, which does not allow us to draw general conclusions about coercive measures. Searching for effects of the two other formal coercive measures should be considered in further research.

Concerning the risks of meta-biases (43), various databases and references of broad reviews on the topic were screened to retrieve any gray literature or unpublished studies that met inclusion criteria. Two conference abstracts were found that presented studies otherwise published and therefore were excluded. Non-English language articles were also included to limit these biases. The risk of selective reporting of outcomes and/or results is certainly elevated as no standard for outcomes exist and registration of trials was not performed except for Huf et al. (44). Overall, these limitations clearly point to the need for more original research on the consequences of coercive measures.

## Conclusions

Effects of seclusion and restraint in adult psychiatry include a wide range of outcomes, and a broad variety of designs has been used to study them. Despite its clear limitations, the identified literature strongly suggests that seclusion and restraint have deleterious physical or psychological consequences. The incidence estimates of PTSD after seclusion or restraint vary from 25% to 47%, which is clearly not negligible, especially for patients with past traumatic experiences. Subjective perception of seclusion and restraint seems to depend on interindividual variability but is largely negative and distressful. No significant differences between them were found in terms of effectiveness or adverse effects. The main negative consequences reinforce the notion that seclusion and restraint should be used with caution and as a last resort method. Patients should be given the opportunity to take part in the decision whenever possible, and their preferences should be taken into account. The therapeutic interaction and relationship could be a main focus for the improvement of effects and subjective perception of coercion. In terms of methodology, studying coercive measures remains difficult and applicability of the evidence is still limited. Well-conducted prospective cohort studies could be more feasible than RCTs for achieving meaningful results on the effects of coercion. In the context of current research on coercion reduction, the study of effects of coercion provides workable baseline data and potential targets for interventions and, thus, a strong motivation for the development of coercion reduction programs.

## Author Contributions

MC contributed to development of the search question and strategies, data collection, and analysis, and to the main part of manuscript redaction. SH participated in development of the search question and strategies. SK supervised advancement of the project and contributed to data selection. OS supervised advancement of the project and participated in development of the search question, strategies, and data extraction. The four authors contributed to manuscript redaction and accepted the present manuscript.

## Funding

This systematic review is part of a research program investigating seclusion and restraint reduction funded by the University Hospital of Geneva.

## Conflict of Interest Statement

The authors declare that the research was conducted in the absence of any commercial or financial relationships that could be construed as a potential conflict of interest.

## Abbreviations

BDI-II, Beck Depression Inventory II; BPRS, Brief Psychiatric Rating Scale; CENTRAL, Cochrane Central Register of Controlled Trials; CINAHL, Cumulative Index to Nursing and Allied Health Literature; DVT, deep vein thrombosis; EUNOMIA, European Evaluation of Coercion in Psychiatry and Harmonization of Best Clinical Practice; PANSS, Positive and Negative Syndrome Scale; PICOS, participants, interventions, comparators, outcomes, study design; PRISMA, Preferred Reporting Items for Systematic Reviews and Meta-Analyses; PROSPERO, International prospective register of systematic reviews; PTSD, post-traumatic stress disorder; RCT, randomized controlled trial; USPSTF, U.S. Preventive Services Task Force.
