# Supplementary material for: Effects of Seclusion and Restraint in Adult Psychiatry: A Systematic Review
Source: Front Psychiatry. 2019 Jul 16;10:491. doi: 10.3389/fpsyt.2019.00491 (PMC6673758; doi:10.3389/fpsyt.2019.00491)
Supplement: Supplementary file 1 [file Table_1.docx]

Supplementary Table 1: Search strategies

| Database | Search |
| --- | --- |
| MEDLINE via Pubmed | ((((((("Mental Disorders"[Mesh] OR "Psychiatry"[Mesh] OR "Hospitals, Psychiatric"[Mesh] OR "Mental Health"[Mesh] OR mental[tiab] OR mentally[tiab] OR psychiatr*[tiab] OR schizophren*[tiab] OR psychoti*[tiab])) AND (((("Restraint, Physical"[Mesh] OR "Coercion"[Mesh] OR "Patient Isolation"[Mesh] OR "Commitment of Mentally Ill"[Mesh])) OR (((Restraint*[tiab] OR Coerci*[tiab] OR Seclusion[tiab] OR Patient Isolation[tiab] OR Patient Immobili*[tiab] OR Compulsor*[tiab] OR Mentally Ill Commitment[tiab] OR Mentally Ill Commitments[tiab] OR Involuntary Commitment[tiab] OR Involuntary Commitments[tiab] OR (involunta*[tiab] NOT movement*[tiab]))) OR ((lock[tiab] OR locked[tiab] OR locking[tiab] OR contained[tiab] OR containement[tiab] OR containment[tiab] OR containment'[tiab] OR containment's[tiab] OR containments[tiab]) AND (door[tiab] OR doors[tiab] OR ward[tiab] OR wards[tiab] OR room[tiab] OR rooms[tiab]))))))) AND ("Outcome Assessment (Health Care)" [Mesh:NoExp] OR "Patient Outcome Assessment" [Mesh:NoExp] OR "Treatment Outcome" [Mesh:NoExp] OR "Beneficence"[Mesh] OR "Risk"[Mesh:NoExp] OR "Risk Assessment"[Mesh:NoExp] OR "Risk Factors"[Mesh] OR "Medical Errors"[Mesh:NoExp] OR "Behavior Control"[Mesh:NoExp] OR "Cooperative Behavior"[Mesh] OR "Patient Acceptance of Health Care"[Mesh:NoExp] OR "Patient Compliance"[Mesh:NoExp] OR "Medication Adherence"[Mesh] OR "Patient Dropouts"[Mesh] OR "Patient Participation"[Mesh] OR "Quality of Life"[Mesh] OR "Clinical Competence"[Mesh] OR "Guideline Adherence"[Mesh] OR "Attitude of Health Personnel"[Mesh:NoExp] OR "Attitude to Health"[Mesh:NoExp] OR "Practice Patterns, Nurses'"[Mesh] OR "Practice Patterns, Physicians'"[Mesh] OR "Professional-Patient Relations"[Mesh:NoExp] OR "Professional Competence"[Mesh:NoExp] OR "Life Style"[Mesh:NoExp] OR "Life Change Events"[Mesh] OR "Dehumanization"[Mesh:NoExp] OR "Prejudice"[Mesh:NoExp] OR "Social Isolation"[Mesh:NoExp] OR "Judgment"[Mesh] OR "Decision Making"[[Mesh:NoExp] OR "Dissent and Disputes"[Mesh:NoExp] OR "Length of Stay"[Mesh] OR ((Outcome [tiab] OR Outcomes [tiab] OR Process [tiab]) AND (Treatment [tiab] OR Assessment [tiab] OR Assessments [tiab] OR Research [tiab] OR Studies [tiab] OR Study [tiab] OR Measures [tiab] OR Measure [tiab] OR Patient-Relevant[tiab] OR Patient Relevant[tiab] OR Rehabilitation[tiab])) OR Effectiveness [tiab] OR Efficiency [tiab] OR Efficacy [tiab] OR Safety [tiab] OR Efficiencies [tiab] OR Commitment Duration [tiab] OR Outpatient Commitment [tiab] OR Beneficence [tiab] OR Benevolence[tiab] OR Nonmaleficence[tiab] OR Risks[tiab] OR Risk Reduction Behavior[tiab] OR Risk Reduction[tiab] OR Harm Reduction[tiab] OR Patient harm[tiab] OR Medical errors[tiab] OR Critical Medical Incidents[tiab] OR Never Events[tiab] OR Behavior control[tiab] OR Cooperative Behavior[tiab] OR Health Care Utilization[tiab] OR Patient Acceptance of Healthcare[tiab] OR Health Care Acceptability[tiab] OR Patient Adherence[tiab] OR Patient Cooperation[tiab] OR Treatment Compliance[tiab] OR Medication Compliance[tiab] OR No Show Patients[tiab] OR No-Show Patient[tiab] OR Patient Involvement[tiab] OR Patient Empowerment[tiab] OR Patient Engagement[tiab] OR Quality of life[tiab] OR Clinical Competency[tiab] OR Clinical Skills[tiab] OR Meaningful Use[tiab] OR Health Personnel Attitudes[tiab] OR Staff Attitude[tiab] OR Staff Attitudes[tiab] OR Health Attitudes[tiab] OR Cultural Care[tiab] OR Nurse's Practice Patterns[tiab] OR Nurse Practice Patterns[tiab] OR Clinical Practice Patterns[tiab] OR Professional-Patient Relations[tiab] OR Professional-Patient Relation[tiab] OR Professional Patient Relationship[tiab] OR Professional Patient Relationships[tiab] OR Human Dignity[tiab] OR Burden of Illness[tiab] OR Respect for Life[tiab] OR Right to Life[tiab] OR Treatment Failure[tiab] OR Professional Competence[tiab] OR Satisfaction[tiab] OR Lifestyle[tiab] OR Life Experiences[tiab] OR Life Experience[tiab] OR Self-Management[tiab] OR Self Care[tiab] OR Patient Reported Outcomes[tiab] OR Patient Reported Outcome[tiab] OR Patient Comfort[tiab] OR Involuntary Psychiatric Treatment[tiab] OR Involuntary Psychiatric Commitment[tiab] OR Dehumanisation[tiab] OR Prejudice[tiab] OR Social Isolation[tiab] OR Loneliness[tiab] OR Social Competence[tiab] OR Social Stigma[tiab] OR Decision making[tiab] OR Approach Behavior[tiab] OR Approach Behaviors[tiab] OR Consensus [tiab] OR Patient Participation[tiab] OR Perception[tiab] OR Death[tiab] OR Mortality[tiab] OR Hospitalization duration [tiab] OR Injury[tiab] OR Insight into illness[tiab] OR Hospital Stay [tiab] OR Hospital Stays [tiab] OR Involuntary medication[tiab]))) AND ((((("randomized controlled trial"[pt] OR "controlled clinical trial"[pt] OR "clinical trials as topic"[mesh] OR "random allocation"[mesh] OR "double-blind method"[mesh] OR "single-blind method"[mesh] OR "clinical trial"[pt] OR "research design"[mesh:noexp] OR "comparative study"[pt] OR "evaluation studies"[pt] OR "follow-up studies"[mesh] OR "prospective studies"[mesh] OR "cross-over studies"[mesh] OR "clinical trial"[tiab] OR ((singl*[tiab] OR doubl*[tiab] OR trebl*[tiab]) AND (mask*[tiab] OR blind*[tiab])) OR placebo*[tiab] OR random*[tiab] OR "control"[tiab] OR "controls"[tiab] OR prospectiv*[tiab] OR volunteer*[tiab])) OR ("cohort studies"[mesh] OR "case-control studies"[mesh] OR "comparative study"[pt] OR "risk factors"[mesh] OR "cohort"[tiab] OR "compared"[tiab] OR "groups"[tiab] OR "case control"[tiab] OR "multivariate"[tiab])) OR (Cross-Sectional Studies [mesh] OR Cross-Sectional Studies [tiab] OR Cross Sectional Studies [tiab] OR Cross-Sectional Study [tiab] OR Cross Sectional Analysis [tiab] OR Cross Sectional Analyses [tiab] OR Disease Frequency Surveys [tiab] OR Disease Frequency Survey [tiab] OR Cross-Sectional Analyses [tiab] OR Cross-Sectional Analysis [tiab] OR Cross-Sectional Survey [tiab] OR Cross Sectional Survey [tiab] OR Cross-Sectional Surveys [tiab] OR Prevalence Studies [tiab] OR Prevalence Study [tiab] OR "Research Support, Non-U.S. Gov't"[pt])))) |
| Embase | ('mental disease'/exp OR 'psychiatry'/exp OR 'mental hospital'/exp OR 'mental health'/exp OR mental:ab,ti OR mentally:ab,ti OR psychiatr*:ab,ti OR schizophren*:ab,ti OR psychoti*:ab,ti) AND ('restraint'/exp OR 'involuntary commitment'/exp OR 'seclusion'/exp OR restraint*:ab,ti OR coerci*:ab,ti OR seclusion*:ab,ti OR compulsor*:ab,ti OR ((patient* NEAR/1 (isolation OR immobili*)):ab,ti) OR (((compulsor* OR involunta* OR legal OR psychiatric OR mental*) NEAR/2 commitment*):ab,ti) OR (((lock OR locked OR locking OR contained OR containement*) NEAR/1 (door* OR ward OR wards OR room OR rooms)):ab,ti) OR (involunta*:ab,ti NOT movement*:ab,ti)) AND ('outcome assessment'/de OR 'treatment outcome'/de OR 'beneficence'/exp OR 'risk'/de OR 'risk assessment'/de OR 'risk factor'/exp OR 'medical error'/de OR 'behavior control'/de OR 'cooperation'/exp OR 'patient attitude'/de OR 'patient compliance'/de OR 'medication compliance'/exp OR 'patient dropout'/exp OR 'patient participation'/exp OR 'quality of life'/exp OR 'clinical competence'/exp OR 'protocol compliance'/exp OR 'health personnel attitude'/de OR 'attitude to health'/de OR 'nursing practice'/exp OR 'clinical practice'/exp OR 'professional-patient relations'/de OR 'professional competence'/de OR 'lifestyle'/de OR 'life event'/exp OR 'dehumanization'/de OR 'prejudice'/de OR 'social isolation'/de OR 'decision making'/exp OR 'conflict'/de OR 'length of stay'/exp OR 'risk factor':ab,ti OR 'patient dropout':ab,ti OR 'conflict':ab,ti OR 'length of stay':ab,ti OR (((outcome* OR process* OR risk* OR harm) NEAR/2 (treatment OR assessment* OR research OR studies OR study OR measure* OR 'patient relevant' OR 'patient relevant' OR rehabilitation OR reduction)):ab,ti) OR effectiveness:ab,ti OR efficienc*:ab,ti OR efficacy:ab,ti OR safety:ab,ti OR ((commitment NEAR/2 (duration OR outpatient)):ab,ti) OR beneficence:ab,ti OR benevolence:ab,ti OR nonmaleficence:ab,ti OR risks:ab,ti OR 'patient harm':ab,ti OR (((medical OR critical) NEAR/2 (error* OR incident*)):ab,ti) OR 'never events':ab,ti OR (((control OR cooperative) NEAR/2 (behavior OR behaviour)):ab,ti) OR ((('health care' OR healthcare) NEAR/2 (utilization OR acceptance OR acceptability)):ab,ti) OR (((patient OR patients OR treatment OR medication OR protocol) NEAR/2 (adherence OR cooperation OR 'no show' OR 'no-show' OR involvement OR empowerment OR engagement OR participation OR compliance OR attitude)):ab,ti) OR 'quality of life':ab,ti OR (((clinical OR professional) NEXT/1 (competency OR competence* OR skill*)):ab,ti) OR 'meaningful use':ab,ti OR (((health OR staff) NEAR/2 attitude*):ab,ti) OR 'cultural care':ab,ti OR (((nurs* OR clinical) NEAR/2 practice NEAR/2 pattern*):ab,ti) OR ((('professional patient*' OR 'professional patient') NEXT/1 relation*):ab,ti) OR 'human dignity':ab,ti OR 'burden of illness':ab,ti OR (((respect OR right) NEAR/2 life):ab,ti) OR (((treatment OR therapy OR medication) NEAR/2 failure):ab,ti) OR satisfaction:ab,ti OR lifestyle:ab,ti OR ((life NEAR/2 (experience* OR event*)):ab,ti) OR 'self management':ab,ti OR ((self NEXT/1 (management OR care)):ab,ti) OR ((patient NEAR/2 reported NEAR/2 outcome*):ab,ti) OR 'patient comfort':ab,ti OR ((involuntary NEAR/2 psychiatric NEAR/2 treatment):ab,ti) OR dehumanisation:ab,ti OR dehumanization:ab,ti OR prejudice*:ab,ti OR 'social isolation':ab,ti OR loneliness:ab,ti OR ((social NEAR/2 (competence* OR stigma)):ab,ti) OR 'decision making':ab,ti OR ((approach NEAR/2 (behavior* OR behaviour*)):ab,ti) OR consensus:ab,ti OR perception:ab,ti OR death:ab,ti OR mortality:ab,ti OR (((hospitalization OR hospital) NEAR/2 (duration OR stay*)):ab,ti) OR injury:ab,ti OR ((insight NEAR/2 illness):ab,ti)) AND ('randomized controlled trial'/exp OR 'controlled clinical trial'/exp OR 'clinical trial (topic)'/exp OR 'randomization'/exp OR 'double blind procedure'/exp OR 'single blind procedure'/exp OR 'clinical trial'/exp OR 'methodology'/de OR 'comparative study'/de OR 'evaluation study'/exp OR 'follow up'/exp OR 'prospective study'/exp OR 'crossover procedure'/exp OR 'cohort analysis'/exp OR 'case control study'/exp OR 'cross-sectional study'/exp OR 'risk factor'/exp OR ((('randomized controlled' OR controlled OR clinical OR comparative OR evaluation OR 'follow up' OR prospective OR crossover OR 'case control' OR 'cross-sectional' OR 'cross sectional' OR 'disease frequency' OR prevalence) NEAR/2 (trial OR trials OR procedure OR procedures OR study OR studies OR analysis OR analyses OR surveys OR survey)):ab,ti) OR (((singl* OR doubl* OR trebl*) NEAR/2 (mask* OR blind*)):ab,ti) OR randomization:ab,ti OR methodology:ab,ti OR 'cohort analysis':ab,ti OR 'risk factor':ab,ti OR placebo*:ab,ti OR random*:ab,ti OR control:ab,ti OR controls:ab,ti OR prospectiv*:ab,ti OR volunteer*:ab,ti OR cohort:ab,ti OR compared:ab,ti OR groups:ab,ti OR multivariate:ab,ti) |
| Google Scholar | (coercion OR Restraint OR seclusion) AND (psychiatric OR psychiatry OR mental health) AND (effect OR safety OR harm OR efficiency OR efficacy OR beneficence OR risk OR mortality OR quality of life OR effectiveness) |
| Web of Science; PsycINFO | (coercion OR Restraint OR seclusion) AND (psychiatric* OR psychiatry OR mental health) AND (clinical trial) |
| Cochrane Central Register of Controlled Trials (CENTRAL); Cumulative Index to Nursing and Allied Health Literature (CINAHL) via EBSCO; Cairninfo; PROSPERO. Clinical.trial.org | (coercion OR Restraint OR seclusion) AND (psychiatric* OR psychiatry OR mental health) |
